# Editorial: Advancements in mass spectrometry for plant metabolomics research

**DOI:** 10.3389/fpls.2026.1866838

**Published:** 2026-05-19

**Authors:** Marisa Maia, Andreia Figueiredo, Vincent Carré

**Affiliations:** 1 Grapevine Pathogen Systems Lab (GPS Lab), Biosystems & Integrative Sciences Institute (BioISI), Faculdade de Ciências da Universidade de Lisboa, Campo Grande, Lisboa, Portugal; 2 Université de Lorraine, Le Laboratoire de Chimie et Physique – Approche Multi-échelles des Milieux Complexes (LCP-A2MC), Metz, France

**Keywords:** bioactive products, liquid and gas chromatography, mass spectrometry imaging, metabolomics, plant improvement, plant metabolic biomarkers

Metabolite synthesis is extremely reactive to any type of changes, providing a real picture of the physiological state of the plant at a given time, allowing researchers to better understand the intricate network of biochemical reactions and pathways within plant cells and tissues.

It is currently known that from all different Kingdoms, plants constitute the group with the greatest diversity of metabolites, possessing around millions of metabolites. However, there is still a long way to go to fully understand metabolome complexity and dynamics in these organisms.

The need for advanced techniques to map this vast metabolite diversity is paramount. Researchers have been applying and developing protocols and methodologies to identify, characterize, and quantify metabolites in different plant systems.

Although different analytical techniques can be employed to study these small molecules, Mass spectrometry has emerged as a power analytical tool for exploring the complex metabolic networks within plants. Recent advancements have significantly enhanced the mass accuracy, resolution, throughput and quantification facilitating deeper insights into plant metabolism.

This Research Topic aimed at gathering novel research on the latest advances in mass spectrometry techniques applied to the study of plants metabolome. Eight original research articles were published focused on innovative mass spectrometry approaches to characterize and quantify metabolites in various plants, ranging from medicinal plants to crops. To achieve this, the authors resort to targeted and non-targeted metabolome profiling approaches, using liquid and gas chromatography, respectively (Dzakovich et al.; Halime et al., 2025; Jiang et al., 2025; Naveena et al., 2025; Wang et al., 2025; Zhong et al.), as well as mass spectrometry imaging techniques, such as Matrix-assisted laser desorption ionization mass spectrometry imaging (MALDI-MSI) (Zhao et al., 2025a, Zhao et al., 2025b) to identify the spatial location of metabolites of interest.

Regarding analytical methods advancements, Dzakovich et al., developed a high-throughput flavonoid extraction method from spinach (*Spinacia oleracea* L.) and established an ultra-high-performance liquid chromatography tandem mass spectrometry (UHPLCMS/MS) method for their analysis. The improved methodology enables a high throughput sample extraction (48 samples in 60 minutes), with a recovery estimated over 100%, allowing time optimization and reduce solvent consumption. This approach enabled the separation and quantification (in 11.5 minutes) of 39 flavonoids unique to spinach by analysing 30 different spinach accessions.

In a similar frame, Jiang et al., 2025 developed an innovative workflow to combine the identification of characteristic fragment ions and neutral losses, obtained through UHPLC-ESI-Q-MS/MS, with an in-house library and GNPS-molecular network to annotate chemical compounds of coumarins. Coumarins are highly produced in *Chimonanthus salicifolius* S. Y., a medicine-food homology plant, widely distributed in Eastern China. The metabolome of different *C. salicifolius* tissues (leaves, branches, roots, seeds, shells of seed and spray-dried powder) were characterized and based on this integrative approach, it was possible to identify around 200 compounds, being more than 60% automatically annotated. Based on the identified compounds, biosynthetic coumarins pathways were predicted. Ultimately, based on the method developed by Jiang et al., 2025 it will be possible to explore other possible applications of *C. salicifolius*, such as in the fields of food and pharmaceutics. Halime et al., 2025 also demonstrated how UHPLC-MS/MS can be used to highlight the metabolic differentiation of harvest residues between two Lupin species (*L. albus* and *L. angustifolius*) to explore species-specific biochemical differences, that define their functional properties and consequently their potential value.

Metabolic profiling (using MS equipment’s coupled with UHPLC/LC and GC), has been widely used to better understand plants and their specific responses to surroundings and environmental conditions as well to pathogens and to highlight their intrinsic properties and value. The study of Zhong et al. presented the dynamics of metabolites in the sand pear cultivar ‘Cuiguan’ upon premature leaf fall. Similarly, to other trees, developed buds of pears remain dormant and only break the dormancy state in spring. However, climatic factors, unexpected disease appearances and early defoliation, have been triggering bud sprouting of pear cultivar ‘Cuiguan’ in autumn leading to significant yield losses on the following year. In this work, Zhong et al. performed an artificial defoliation treatment in pear cultivar “Cuiguan” trees and compared to a control group. The authors applied LC-MS/MS and were able to identify a total of 1533 differentially accumulated metabolites (898 in positive ion mode and 635 in negative ion mode) associated with bud release and subsequent blooming in autumn. Compared to control buds, the defoliation treated trees showed an early response through the significant reduction of sugars and amino acids.

Wang et al. showed how different elevation (4300m, 4600m and 5000m) and slope orientation (south vs north) can impact the metabolome of *Phlomoides rotata*, a traditional Tibetan medicinal herb through LC-MS. The results showed that altitude mainly increase the accumulation of flavonoids, likely related to adaptative responses to UV radiation and oxidative stress. The difference in slope orientation led to 17 differently accumulated metabolites, particularly glycerophospholipids. The authors reported that these results may be related to microclimate differences in temperature and moisture.

Regarding pathogen interaction, Naveena et al., 2025 studied the potential of over 40 endophytic bacteria with antagonistic activities against *R. solani*. *Rhizoctonia solani*, is the rice sheath blight disease etiological agent. This disease affects the rice production worldwide with significant yield losses every year, requiring intensive fungicide application. To develop new disease management strategies, the authors tested the biocontrol effect of different endophytic bacteria, identifying *Bacillus velezensis* B13 as the most effective one (highest mycelial inhibition against *R. solani*). The authors resort to GC-MS to analyse the bioactive compounds produced by strain B13 that inhibited *R. solani* growth and identified 12 metabolites that presented antifungal and antimicrobial capabilities. The authors validated their results through an *in vivo* study, in which both seed treatment and foliar spraying of *B. velezensis* B13 in rice plants resulted in a disease reduction compared to the control.

Mass spectrometry imaging (MSI) is currently one of the most top edge mass spectrometry techniques which is being applied to plant tissues. The principle of MSI is based on the measurement of mass spectra by scanning numerous designated areas on the surface of a sample using a spatially controlled ionization device. By analysing variations in signal intensity across the mass spectra, it is possible to determine the spatial distribution of multiple compounds during a single imaging experiment, thereby providing a better understanding of their tissue-specific roles and mechanisms of action. Its potential is fully illustrated by the study of Mengwei Zhao et al. where the authors applied matrix-assisted laser desorption/ionization mass spectrometry imaging (MALDI-MSI) to reveal the spatial distribution of metabolites during three developmental stages of Ziziphus jujube Mill. var. spinosa fruit (Zhao et al., 2025a). The seed of this plant is highly popular in China and it is known for its food and pharmaceutical properties. MALDI-MSI was used to analyse this fruit in white maturity, firm ripening and full ripening stages. Twenty-five bioactive metabolites were identified, ranging from different metabolic classes such as flavonoids, terpenoids and alkaloids, and their spatial distribution were analysed in the three developmental stages. Through this technique, the authors could correlate the increase in accumulation of these bioactive metabolites with the ripening of the fruit (Zhao et al., 2025a). Similarly, Zhao et al. also applied MALDI-MSI to the study of a fruit. In this study, the authors wanted to better understand the differences in pigment accumulation in distinct colour regions (red and yellow regions) in *Yanzhihong* apricot (*Prunus armeniaca* L.). The results revealed that sucrose and carotenoids are highly accumulated in the red region of the apricot while fructose, ascorbic acid, malic acid and citric acid have significantly higher levels in the yellow region.

The contributions in this Research Topic underscore the pivotal role of advanced mass spectrometry techniques in advancing plant metabolomics. Through innovative analytical and imaging approaches, these studies enhance our ability to identify, quantify, and localize metabolites across diverse plant systems. While significant progress has been made, the vast complexity of plant metabolomes highlights the ongoing need for methodological innovation and deeper exploration.
